# Local transplantation of adipose-derived stem cells has a significant therapeutic effect in a mouse model of rheumatoid arthritis

**DOI:** 10.1038/s41598-020-60041-2

**Published:** 2020-02-20

**Authors:** Hideki Ueyama, Tadashi Okano, Kumi Orita, Kenji Mamoto, Masaaki Ii, Satoshi Sobajima, Hideki Iwaguro, Hiroaki Nakamura

**Affiliations:** 1 Department of Orthopedic Surgery, Osaka City University Graduate School of Medicine, Osaka, Japan; 2 Sobajima Clinic, Osaka, Japan; 3 Nanobridge LLC, Osaka, Japan

**Keywords:** Drug delivery, Mesenchymal stem cells

## Abstract

Adipose-derived stem cells (ADSCs) have anti-inflammatory and regenerative properties. The purpose of this study was to investigate the effect of locally administered ADSCs in a rheumatoid arthritis (RA) mouse model. In an *in vivo* experiment, single-cell ADSCs and three dimensionally-cultured ADSC spheroids were injected intra-articularly into the knees of RA model mice and histologically assessed. Marked improvement of synovial inflammation and articular cartilage regeneration was found in ADSC-treated mice. Proliferation, migration, and apoptosis assays of synovial fibroblasts incubated with single-cell and spheroid ADSCs were performed. The expression levels of total cytokine RNA in ADSC single cells, spheroids, and ADSC-treated inflammatory synovial fibroblasts were also evaluated by quantitative reverse transcription PCR. ADSCs suppressed the proliferation and migration of activated inflammatory cells and downregulated inflammatory cytokines. *TSG-6* and *TGFβ1* were significantly upregulated in ADSCs compared to controls and *TGFβ1* was significantly upregulated in ADSC spheroids compared to single cells. The apoptosis rate of ADSC spheroids was significantly lower than that of single-cell ADSCs. These results indicated that intra-articular administration of ADSC single cells and spheroids was effective in an RA mouse model, offering a novel approach for the development of effective localized treatments for patients with RA.

## Introduction

Rheumatoid arthritis (RA) is an autoimmune disease characterized by synovial inflammation and degeneration of articular cartilage and bone. In previous decades, systemic therapies, including methotrexate and biological disease-modifying antirheumatic drugs, significantly improved the treatment of synovial inflammation^1^. Given that RA is a systemic disease, the focus has been to develop systemic treatments for it^2^; however, swelling persists in some joints with such treatments and the application of systemic treatment is limited due to adverse, off-target effects. Therefore, localized treatment methods are important for treating RA. Corticosteroid injections are the most widely used localized treatment for RA. Although it reduces RA-associated synovitis, repeated intra-articular corticosteroid injections can lead to the degeneration of articular cartilage^3^. Other adverse effects of corticosteroids include septic arthritis, local tissue atrophy, tendon rupture, and hyperglycaemia. Consequently, the development of safe, effective, and tissue-regenerating localized therapies must be explored.

Recent studies in regenerative medicine have used mesenchymal stem cells (MSCs) derived from somatic tissues such as bone marrow, muscle, blood, and adipose tissue^4,5^. MSCs exert anti-inflammatory effects, modulate the immune response, and modify the local microenvironment; furthermore, studies have reported their therapeutic effects for the treatment of inflammatory and autoimmune diseases^6,7^. Considering the accessibility and ethical issues associated with the use of MSCs, adipose-derived stem cells (ADSCs) have recently emerged as attractive alternatives for stem cell therapy. ADSCs reportedly exert marked immunomodulatory and protective effects in animal models of acute graft versus host disease and animal models of arthritis^8,9^. Previous ADSC studies on the treatment of RA have explored systemic methods^10–13^ involving localized treatment using only MSCs from other materials^14,15^. An effective localized treatment has yet to be demonstrated in a mouse model of RA using ADSCs. In addition, three-dimensional (3D) culturing reportedly increases the therapeutic effects of MSCs *in vitro*^16^. These effects have not yet been assessed in a mouse model of RA.

Sakaguchi *et al*. used a mouse model (SKG model) of inflammatory polyarthritis established with a spontaneous point mutation in Zap70 in BALB/c mice^17^. SKG mice develop T cell-mediated chronic autoimmune polyarthritis, characterized by joint inflammation and articular cartilage degeneration^18,19^ that is strikingly similar to the clinical progression of RA in humans.

We hypothesized that localized therapy using ADSC single cells and 3D cultured ADSC spheroids might be effective for the treatment of RA. The aim of this study was to assess the effects of the local administration of ADSC single cells and ADSC spheroids in a mouse model of inflammatory RA.

## Results

### Arthritis induction in a mouse model of rheumatoid arthritis

**Figure 1:**
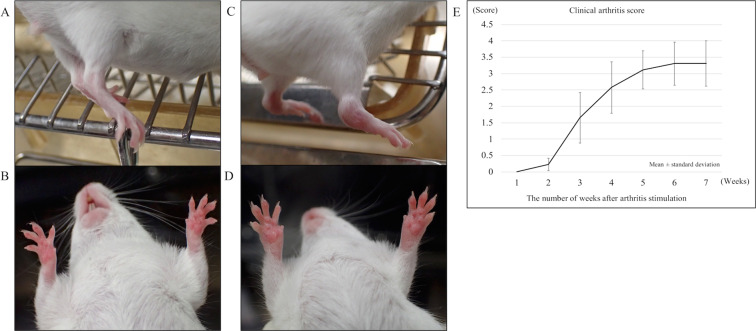
Laminarin-induced arthritis in SKG mice. (**A**,**B**) Representative images of healthy joints. (**C**,**D**) Representative images of joint inflammation and swelling. (**E**) Changes in clinical arthritis scores over time in SKG mice after adjuvant administration. The severity of inflammation correlated with the clinical score. The score increased with time. Arthritis scores of 74 mice were assessed before histological experiments to confirm inflammation.

Arthritis was induced in female SKG mice via an intraperitoneal injection of laminarin (30 mg per mouse). Polyarticular arthritis originated from the interphalangeal joints several weeks after adjuvant injection. SKG mice with a clinical arthritis score between 3 and 4 (active inflammation) were used for subsequent experiments (Fig. 1).

### Adipose-derived stem cells reduced joint inflammation in a mouse model of rheumatoid arthritis

**Figure 2:**
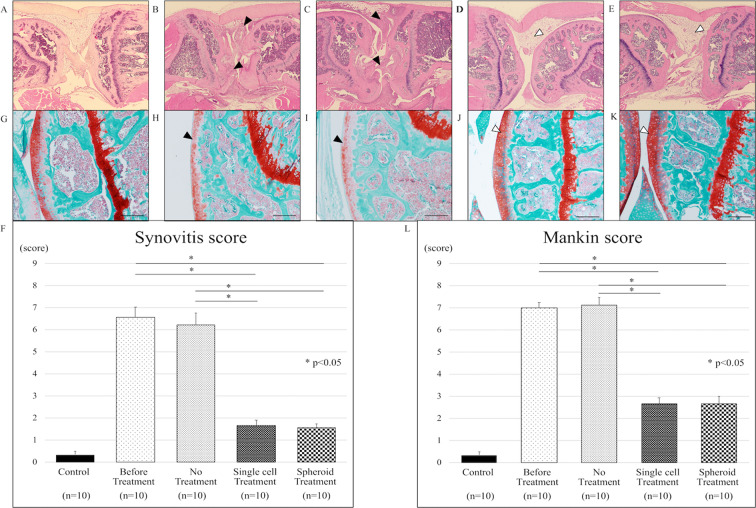
Adipose-derived stem cells reduce joint inflammation in a mouse model of rheumatoid arthritis. Collectively, 50 mice were divided into 5 groups of 10 mice each. (**A**–**E**) Haematoxylin and eosin (H&E) staining of representative knee tissue sections. (**A**) Representative image of healthy knee tissue (control group). (**B**) Inflammatory cell infiltration into the synovium of the knee joint of SKG mice with active inflammation (black arrow-heads). (**C**) The inflammatory cell infiltration into synovium persists in the knee joint without treatment (black arrow-heads). (**D**) Knee inflammation improved upon intra-articular injection of ADSC single cells (white arrow-head). (**E**) ADSC spheroid injection into the knee joint decreased inflammation (white arrow-head). (**F**) Synovitis scores for tissue sections in indicated treatment groups. ADSC single cell and ADSC spheroid treatment significantly improved the synovitis score compared to the untreated group (p < 0.05). (**G**–**K**) Safranin-O staining of representative knee tissue sections. (**G**) Representative image of healthy knee tissue (control group). (**H**) Safranin-O staining showing a moderate reduction in the articular cartilage of SKG mice with active inflammation (black arrow-head). (**I**) Staining intensities of untreated articular cartilage were reduced (black arrow-head). (**J**,**K**) Staining intensities of articular cartilage treated via intra-articular injection of ADSC single cells or ADSC spheroids were enhanced (white arrow-head). (**L**) Mankin scores for tissue sections in indicated treatment groups. Mankin scores improved significantly in the treated groups (ADSC single cells and ADSC spheroids) compared to the untreated group (p < 0.05). Scale bars represent 200 μm in all panels.

We evaluated the morphology and immune cell infiltration in knee tissue sections. Knee tissue sections of SKG mice without laminarin administration did not show inflammation (Fig. 2A). SKG mice administrated with laminarin exhibited severe synovitis, accompanied by massive sub-synovial infiltration of neutrophils, lymphocytes, and macrophages (Fig. 2B). Without ADSC treatment, inflammation persisted (Fig. 2C). After intra-articular injection of ADSCs, the thickness of the synovium and inflammatory cell infiltration in the knee were reduced in the mice with laminarin-induced arthritis (Fig. 2D,E). ADSC treatment (“Single cell” and “Spheroid”) significantly reduced synovitis scores compared to no treatment (“No”) (Fig. 2F). Synovitis scores were 0.33 ± 0.17 in control mice, 6.56 ± 0.48 in mice with laminarin-induced arthritis before treatment, 6.22 ± 0.52 in mice with laminarin-induced arthritis with no treatment, 1.67 ± 0.24 in mice with laminarin-induced arthritis after treatment with ADSC single-cell suspensions (p < 0.05), and 1.55 ± 0.18 in mice with laminarin-induced arthritis after treatment with ADSC spheroids (p < 0.05).

### Adipose-derived stem cells regenerated damaged articular cartilage in a mouse model of rheumatoid arthritis

We evaluated safranin-O staining in knee tissue sections of control and treated SKG mice. The articular cartilage in control mice did not display cellular or tissue irregularities (Fig. 2G). SKG mice administrated with laminarin exhibited reduced safranin-O staining intensity of articular surfaces (Fig. 2H). In ADSC-untreated mice, the reduction of safranin-O staining intensity at articular surfaces persisted (Fig. 2I). On the other hand, knee tissue in ADSC-treated SKG mice retained safranin-O staining at articular surfaces (Fig. 2J,K). ADSC treatment significantly reduced Mankin scores in ADSC-treated mice vs. untreated mice with laminarin-induced arthritis (Fig. 2L). The Mankin scores were 0.33 ± 0.15 in control mice, 7 ± 0.23 in mice with laminarin-induced arthritis before treatment, 7.11 ± 0.35 in mice with laminarin-induced arthritis and no treatment, 2.67 ± 0.26 in mice with laminarin-induced arthritis after treatment with ADSC single-cell suspensions (p < 0.05), and 2.67 ± 0.34 in mice with laminarin-induced arthritis after treatment with ADSC spheroids (p < 0.05).

### Adipose-derived stem cells suppressed immune cell infiltration in a mouse model of rheumatoid arthritis

**Figure 3:**
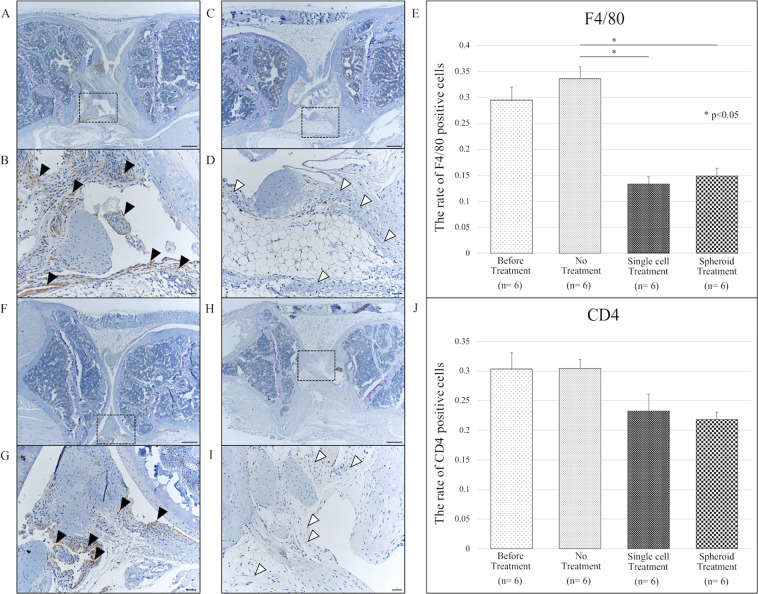
Adipose-derived stem cells reduce immune cell infiltration in a mouse model of rheumatoid arthritis. Altogether, 24 mice were divided into 4 groups of 6 mice each. (**A**–**D**) Representative images of F4/80 staining to detect macrophage infiltration in intra-articular synovial sections of mice in indicated treatment groups. (**A**,**B**) F4/80-positive cells infiltrated into the intra-articular synovium of SKG mice with laminarin-induced arthritis. Black arrow-heads pointed F4/80-positive cells. (**C**,**D**) F4/80-positive cells decreased in the knees of adipose-derived stem cell (ADSC)-treated mice. White arrow-heads pointed synovium. (**E**) The rate of F4/80-positive cells decreased significantly in groups treated with ADSC single cells and ADSC spheroids (*p < 0.05). (**F**–**I**) Representative images of CD4 staining to detect helper T cell infiltration in intra-articular synovial sections. (**F**,**G**) CD4 positive cells infiltrated into the intra-articular synovium of SKG mice with laminarin-induced arthritis. Black arrow-heads pointed CD4 positive cells. (**H**,**I**) CD4 positive cells in the intra-articular synovium decreased after ADSC treatment. White arrow-heads pointed synovium. (**J**) The rate of CD4 positive cells in the knee decreased slightly but not significantly in the ADSC single cell or ADSC spheroid treatment groups. Scale bars represent 200 μm. CD; cluster of differentiation.

To assess macrophage and helper T cell infiltration in murine synovial tissue, we performed immunostaining for F4/80 and CD4, respectively. In mice with laminarin-induced arthritis, treatment with both ADSC single-cell suspensions and ADSC spheroids significantly decreased macrophage infiltration vs. no treatment (p < 0.05) (Fig. 3A–E). There were no significant differences in CD4 positive cells in any of the groups (Fig. 3F–J).

### Adipose-derived stem cells suppressed migration and proliferation of synovial fibroblasts *in vitro*

**Figure 4:**
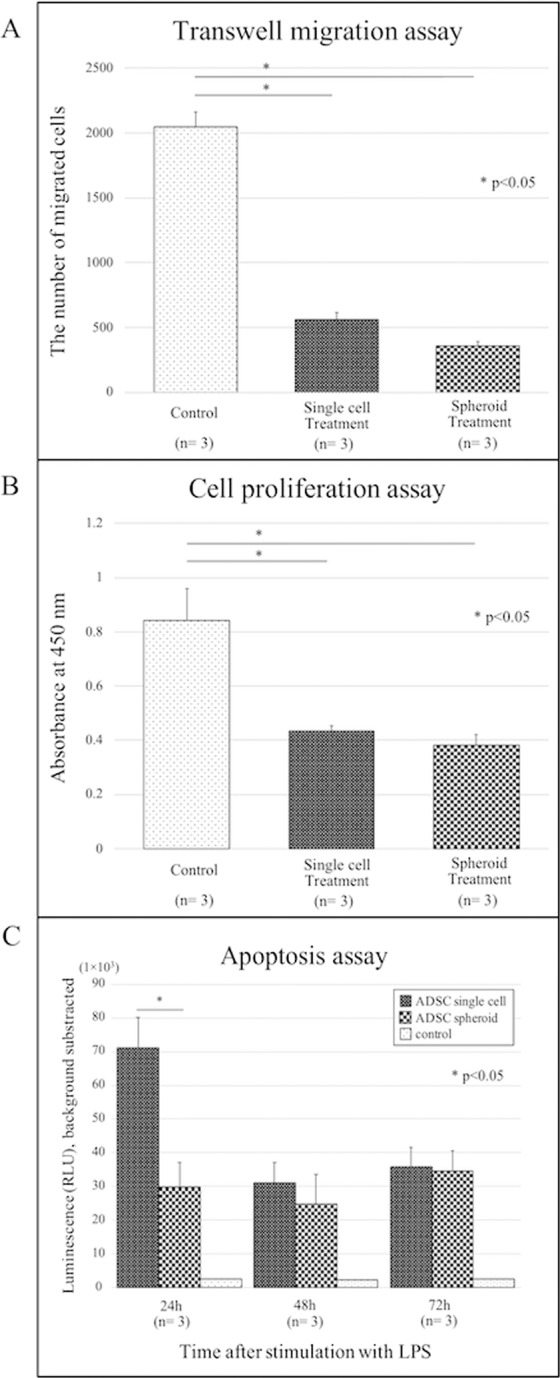
Adipose-derived stem cells inhibit the migration and proliferation of synovial fibroblasts. (**A**) Transwell migration assay. Synovial fibroblasts were seeded in the top well of transwell chambers with 5-μm pore membranes. Adipose-derived stem cells (ADSC) single cells, ADSC spheroids, or no cells (“Control”) were seeded in the bottom chambers. After 16 h, the number of synovial fibroblasts that migrated into the lower surface of the transwell membrane was assessed. Data represent the mean ± SD of 3 independent experiments, (*p < 0.05). (**B**) Proliferation as measured by difference in optical density (OD) using Cell Counting Kit-8. ADSC single cells, ADSC spheroids, or no cells (“Control”) were seeded in the top wells of transwell chambers with 0.4-μm pore membranes. Synovial fibroblasts were seeded in the bottom chambers. After 50 h, proliferation of synovial fibroblasts was assessed as the difference in OD at 450 nm among each co-culture condition for ADSC single cell, ADSC spheroid, or non-treatment groups. Data represent the mean ± SD of 3 independent experiments, (*p < 0.05). (**C**) The progression of apoptosis of ADSC single cells and spheroids was compared at each time point (24, 48, and 72 hours) after stimulation with liposaccharide (LPS) using a Caspase-Glo 3/7 Assay Kit. In the early phase (24 h), apoptosis of spheroids was suppressed compared to that of single cells (*p < 0.05).

Synovial cell migration was observed from the upper to the lower chamber through a transwell membrane. This cell migration was observed for all specimens and evaluated via fluorescence microscopy. Cell migration was significantly reduced in both ADSC single cell-treated and ADSC spheroid-treated synovial fibroblasts compared to that in the control group. DAPI-positive cells were counted, with 2044 ± 117 in the control group, 561 ± 54 in the ADSC single cell-treated group (p < 0.05), and 358 ± 35 in the ADSC spheroid-treated group (p < 0.05) (Fig. 4A).

Synovial cell proliferation was quantified using a CCK-8 kit based on the OD value. The OD value of the chamber of cultured synovial cells was 0.84 ± 0.12 for the control group, 0.43 ± 0.02 for the ADSC single cell-treated group, and 0.38 ± 0.04 for the ADSC spheroid-treated group (Fig. 4B).

### Apoptosis of ADSC spheroids was suppressed compared to that of ADSC single cells

The apoptotic rate of ADSC spheroids was significantly lower than that of ADSC single cells 24 h after the baseline time (p < 0.05) (Fig. 4C). There was no significant difference in luminescence 48 h and 72 h after the baseline time in both ADSC types. This result showed that apoptosis of ADSC spheroids might be suppressed compared to that of ADSC single cells in the early phase of seeding into the lesion site.

### Adipose-derived stem cell spheroids expressed higher levels of *TSG-6* and *TGFβ1* than did single-cell cultures

**Figure 5:**
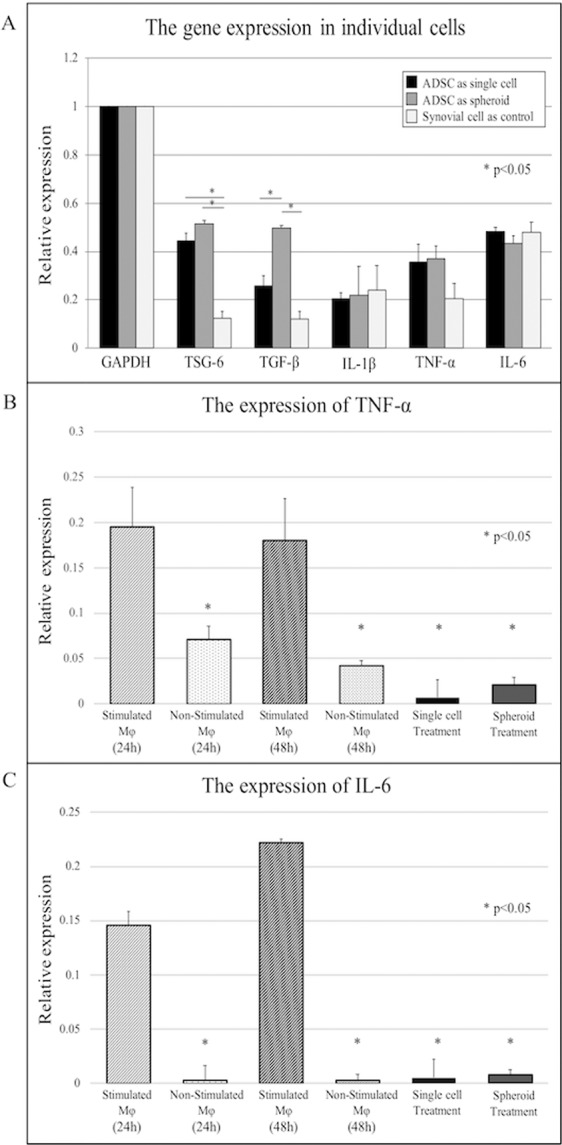
Adipose-derived stem cells express *TSG-6* and inhibit *TNF-α* and *IL-6* of stimulated macrophages. (**A**) Relative total RNA expression levels of *TSG-6*, *TGFβ1*, *IL-1β*, *TNF-α*, and *IL-6* were assessed in synovial cells (control), adipose-derived stem cell (ADSC) single cells, and ADSC spheroids by RT-qPCR. Expression levels were normalized to GAPDH. The expression levels of *TSG-6* and *TGF-β* in ADSC single cells and ADSC spheroids were higher than in the control. (**B**,**C**) Relative total RNA expression levels of *TNF-α* (**B**) and *IL-6* (**C**) were assessed in unstimulated macrophages, stimulated macrophages, and stimulated macrophages after treatment with ADSC single cells and ADSC spheroids. Expression levels were normalized to GAPDH. The expression levels of *TNF-α* and *IL-6* were significantly lower in ADSC single cell and ADSC treatment groups than in the non-treatment group (*p < 0.05).

We evaluated the total RNA levels of *TSG-6*, *TGFβ1*, *IL-1β*, *TNFα*, *IL-6*, and *GAPDH* in synovial fibroblasts (controls), ADSC single cells, and ADSC spheroids (Fig. 5A). *TGFβ1* was expressed at significantly higher levels in ADSC cells and spheroids compared to controls (p < 0.05). *TGFβ1* total RNA was significantly upregulated in ADSC spheroids compared to ADSC single cells. The expression levels of *IL-6*, *TNFα*, and *IL-1β* were not significantly different among the groups.

### Stimulated macrophages expressed higher levels of *IL-6* and *TNFα* than did unstimulated macrophages, and treatment with adipose-derived stem cells inhibited the stimulation-related upregulation

We evaluated the total RNA levels of *TNFα*, *IL-6*, and *GAPDH* in unstimulated macrophages, macrophages stimulated with LPS, and stimulated macrophages after the addition of ADSC single cells or ADSC spheroids. *IL-6* and *TNFα* total RNA levels were significantly higher in stimulated compared to unstimulated macrophages (p < 0.05) and LPS-induced elevation of *IL-6* and *TNFα* levels was significantly inhibited by the addition of ADSC single cells or spheroids (p < 0.05) (Fig. 5B,C).

## Discussion

In this study, we found that localized injection of ADSC cells and spheroids reduces intra-articular inflammation and regenerates damaged cartilage in a mouse model of RA. We also demonstrated the inhibitory effects of ADSC cells and spheroids on synovial fibroblasts and activated macrophages *in vitro*. *TSG-6* and *TGFβ1* total RNA expression levels were elevated in ADSC single cells and spheroids compared to synovial fibroblasts, with ADSC spheroids expressing higher levels of *TGFβ1* than their single-cell counterparts. Furthermore, co-culturing activated macrophages with ADSC cells and spheroids suppressed the LPS-induced elevation of *TNFα* and *IL-6*.

Both ADSC cells and spheroids suppressed inflammation in a mouse model of RA. Based on our *in vitro* data, this therapeutic effect may be due to the elevation of anti-inflammatory cytokines and *TSG-6* as well as the suppression of migration or proliferation of activated inflammatory cells. *TSG-6* exhibits anti-inflammatory effects by inhibiting *TLR4/NF-κB* signalling and *STAT1* and *STAT3* activation^20,21^. Additionally, ADSCs decrease the DNA binding activity of *NF-κB* and switch M1 macrophage activity to anti-inflammatory M2 macrophage activity^22,23^. These findings support the results we obtained in our study. These functions of ADSCs may contribute to the suppression of active inflammation.

Although spheroids may offer certain treatment advantages, there was no statistically significant difference between treatment using ADSC single cells and spheroids in mice. *In vitro*, the expression level of *TGFβ1* in ADSC spheroids was twice that in single-cell cultures. Additionally, our results showed that apoptosis in ADSCs was suppressed in the form of spheroids rather than in single cells. *TGFβ1* is positively correlated with regenerative effects^24^. The *TGFβ* family of proteins promotes *Smad2* signalling. Crosstalk between *TGFβ* and bone morphogenic protein (*BMP*) pathways contribute to the formation and maintenance of articular cartilage^25,26^. In the present study, the expression of *TGFβ1* was higher in spheroids than in single cells of ADSCs. This may be attributed to cell-to-cell interaction, changes in the intracellular microenvironment, or increased secretion of cytokines from cell spheroids^27–29^. Moreover, from a clinical perspective, spheroids may have some advantages, as they promote the migration of large numbers of cells or aggregates to the lesion site. In previous studies, many stem cell-based therapies have shown dose-dependent effectiveness^30,31^. Therefore, higher numbers of stem cells delivered locally to the lesion may promote a favourable therapeutic effect. Based on the results of our apoptosis assay, it can also be said that cell spheroids may also extend the duration of a cell’s life. *In vivo*, single-cell suspensions tend to experience a more programmed cell death than cell spheroids^32^.

The limitations of this study include the following. First, mouse ADSCs, macrophages, and human synovial cells were used for *in vitro* analysis. It would be more appropriate to use human ADSCs from a clinical perspective. However, previous studies on ADSCs have demonstrated their effectiveness across several species^33–35^. Second, the severity of RA in our mouse model was evaluated on the basis of generalized clinical scoring. Although these scores correlate well with arthritis severity, slight differences have been observed between individuals^17^. Lastly, the molecular mechanisms by which ADSCs exert their anti-inflammatory and regenerative effects after intra-articular injection have not yet been characterized. A previous study reported that intra-articularly injected ADSCs migrate to the synovium in an animal model of osteoarthritis^15^. Hence, similar dynamics may also be expected in our animal model.

In summary, we demonstrated the therapeutic effects of local administration of ADSC single cells and spheroids in a model mouse of RA. The suppression of synovial cell and macrophage function and the upregulation of TSG-6 and TGFβ1 in mouse ADSCs could be the putative underlying mechanism. This novel localized therapy using ADSC spheroids may offer a promising therapeutic strategy for the treatment of RA.

## Methods

### Animals

Female SKG and BALB/c mice were obtained from CLEA Japan, Inc. (Tokyo, Japan). Mice were housed in the Osaka City University animal facility under climate-controlled conditions at a constant temperature and humidity under a 12-hour light/dark cycle with *ad libitum* access to standard laboratory chow and water. All experiments were conducted in accordance with institutional guidelines.

### Induction of arthritis and evaluation of clinical scores

Ten to twelve-week-old SKG mice were treated with 30 mg laminarin (ASB-00012020; Wako, Tokyo, Japan), as described previously^36^. Briefly, laminarin was suspended in Dulbecco’s phosphate buffered saline (D-PBS 045-29795; Wako) to a concentration of 10 mg/mL; 3 mL were injected intraperitoneally on day 0. Arthritis developed 4–6 weeks after injection. Clinical scores were recorded weekly (as described previously^17^) based on the following observations: 0, no swelling or redness; 0.1, swelling or redness in the digits; 0.5, mild swelling and/or redness in the wrists or ankle joint; and 1, severe swelling of larger joints. Arthritis scores of 74 mice were assessed before histological experiments.

### Harvesting of adipose tissue and adipose stem cell isolation and culture

Ten to twelve-week-old female BALB/c mice were anesthetized via subcutaneous administration of a solution of ketamine and xylazine (5 mg/mouse) and euthanized. Adipose tissue was harvested from the inguinal region and used in all experiments as subcutaneous white adipose tissue. ADSCs were isolated from adipose tissue based on a previously described procedure^37^ with slight modifications^35,38^. Briefly, adipose tissue harvested from mice subcutaneous area was washed in PBS, minced, and digested in 5 mL of type VIII collagenase (1 mg/mL in 1% BSA/HBSS; Life Technologies Japan, Tokyo, Japan) for 40 min at 37 °C using a Bioshaker BR15 (TAITEC, Tokyo, Japan) in accordance with the manufacturer’s instructions. The digested tissue was filtered through a 40-μm cell strainer (BD Falcon, Tokyo, Japan) and centrifuged at 250 × g at 20 °C for 10 min. Pelleted cells were suspended in 5 mmol/L EDTA/PBS and layered over an equal volume of 0.83 g/L NH_4_Cl solution (Wako). After further centrifugation at 250 × g at 20 °C for 10 min, mononuclear cells were harvested from the gradient interface and used as freshly isolated ADSCs for the experiments. The isolated mouse ADSCs from this protocol expressed characteristic biomarkers of stem cells (e.g., Sca-1) and mesenchymal cells (e.g., CD44) in the previous report^38^. Cells were cultured in DMEM (Wako) supplemented with 10% foetal bovine serum (FBS), dissociated at approximately 70% confluence (TryLE Express 1×; Life Technologies Japan, Tokyo, Japan), and replated at 1000–3000 cells/cm^2^. Mouse ADSCs were cultured and used between passages 4 and 6.

### Three-dimensional culturing of adipose-derived stem cell spheroids

Mouse ADSCs were prepared as 1 × 10^5^/mL cell suspensions. Single drops of the suspension (100 μL per drop containing approximately 1 × 10^3^ cells) were pipetted onto the covers of 96-well V bottom plates (MS-9096U; Sumitomo Bakelite, Tokyo, Japan). ADSCs were cultured inversely for 48 h in an incubator at 37 °C with 5% CO_2_. Spheroid formation/morphology was confirmed microscopically before their use in experiments.

### Intra-articular injection of adipose-derived stem cell single-cell suspensions and spheroids

ADSCs were injected bilaterally into the knees of SKG mice with inflammation scores of 3–4 points, as described previously^39^. In order to inject certainly, mice were anesthetized and skin incisions were made to expose the articular capsules completely, but the joint capsule was not open. An orthopaedic surgeon injected 1.5 × 10^4^ cells/knee (10 μL/knee) of an ADSC single-cell suspension into the intra-articular space with a 29G needle microsyringe (Hamilton, Reno, NV, USA) under a microscope (SZ61; Olympus, Tokyo, Japan); 15 ADSC spheroids (1 × 10^3^ cells/spheroid, 10 μL/knee) were injected via the same procedure. In the control group, PBS (10 μL/knee) was injected. Clinical scores were recorded each week after intra-articular injection.

### Preparation of knee tissue samples

Collectively, 74 SKG mice underwent intra-articular injection and were histologically assessed. Fifty mice were used for Harris haematoxylin and eosin (H&E) or Safranin-O stains and 24 mice were used for immunohistochemistry. Knee tissues from SKG mice were harvested 2 weeks after intra-articular ADSC injection, fixed in 4% paraformaldehyde overnight, decalcified in 0.5 M EDTA (pH 7.5) for 7 days, and embedded in paraffin. The tissues were cut into 4-μm sagittal sections, mounted on slides, deparaffinized with xylene, and dehydrated with graded ethanol solutions prior to histological analysis or immunohistochemistry. After we confirmed the reproducibility of this experimental approach, we performed the histological data analyses on 50 mice to evaluate H&E and safranin-O stains. Moreover, we performed the immunohistochemistry data analyses on different 24 mice treated at the different time under the same condition.

### Histological analysis of knee tissue

Tissue sections were stained with H&E, safranin-O, and fast green to detect the proteoglycan content. Inflammation and cartilage damage were scored as described previously using the synovitis score and the Mankin score^40,41^. Particularly, 50 mice were used for this experiment. Ten mice without inflammatory stimulation were used as the control group. Ten mice with inflammatory stimulation and no intra-articular injection were regarded as the before-treatment group. Thirty mice with inflammatory stimulation that underwent treatments were divided as follows: no treatment group (n = 10, PBS injection), single-cell treatment group (n = 10, ADSC single-cell injection), and spheroid treatment group (n = 10, ADSC spheroid injection).

### Immunohistochemistry

Following deparaffinization and dehydration, tissue samples were washed in PBS. Sections were incubated at 95–100 °C for 30 min in Target Retrieval solution 10× (Dako, Santa Clara, CA, USA) for antigen retrieval, then incubated with 3% H_2_O_2_ to block intrinsic peroxidase activity. Non-specific antibody-binding sites were blocked using 3% normal rabbit serum (Nichirei, Tokyo, Japan). Specimens were incubated with appropriately diluted primary mouse anti-F4/80 and anti-CD4 antibodies at 4 °C overnight. After three PBS washes, specimens were incubated in the secondary antibody (biotin-labelled rabbit anti-rat IgG; Vector Labs, Burlingame, CA, USA) for 60 min at 20 °C. Staining was detected with 3,3′-diaminobenzidine (DAB; Nichirei) and haematoxylin was used for counterstaining. Slides were observed under a light microscope (SZ61; Olympus)^42^. In this experiment, 24 mice were assessed. Six mice with inflammatory stimulation and no intra-articular injection were regarded as the before-treatment group. Eighteen mice with inflammatory stimulation that underwent treatments were divided as follows: no treatment group (n = 6, PBS injection), single-cell treatment group (n = 6, ADSC single-cell injection), and spheroid treatment group (n = 6, ADSC spheroid injection).

### Harvesting of synovial tissue and isolation of synovial fibroblasts

Synovial membranes were harvested during arthroplasty from an RA patient at our institution with active inflammation who provided written informed consent to participate in the study. Primary RA synovial fibroblasts were isolated based on a previous procedure that describes the successful isolation of human RA synovial fibroblasts with minor modifications^43^. Briefly, synovial tissues from RA patients were stored in 70% ethanol at the operation room; in the laboratory, the samples were minced and incubated for 4 h with 2.5 mg/mL type I collagenase (Sigma Aldrich, St. Louis, MO, USA) in Roswell Park Memorial Institute (RPMI) 1640 (Sigma-Aldrich) at 37 °C in 5% CO_2_. The digested tissue was filtered through a 40-μm cell strainer (BD Falcon, Tokyo, Japan) and centrifuged at 1000 rpm at 20 °C for 10 min. Pelleted cells were suspended in 5 mmol/L EDTA/PBS and layered over an equal volume of 0.83 g/L NH_4_Cl solution (Wako). After further centrifugation at 1000 rpm at 20 °C for 10 min, cells were resuspended in RPMI supplemented with 10% FBS, 2 mM L-glutamine, 100 U/mL penicillin, and 100 mg/mL streptomycin and plated onto 100-mm cell culture plates. At approximately 70% confluence, non-adherent cells were removed by medium change, and adherent cells were dissociated (TryLE Express 1×; Life Technologies Japan) and plated at 1000–3000 cells/cm^2^ as human synovial fibroblasts. Human synovial fibroblast cells were cultured and used between passages 4 and 6.

### Transwell migration assay

To evaluate the effects of ADSCs on cell migration, ADSCs and synovial cells were co-cultured according to the standard protocol for transwell migration assays^44^. Briefly, using 12-well transwell plates with a 5-μm pore membrane (3421; Corning, Corning, NY, USA), synovial cells (5 × 10^4^ cells/well) were seeded in the upper chambers in serum-free media; ADSCs (5 × 10^4^ cells/well) or 20 ADSC spheroids/well (2.5 × 10^3^ ADSC per spheroid) were seeded in the lower chambers in media supplemented with 10% FBS. After 16 h at 37 °C, cells were removed from the upper surface of the membrane. Cells that migrated to the lower surface of the membrane were stained with 4′,6-diamidino-2-phenylindole (DAPI) and three microscopic fields were photographed under a fluorescence microscope (BZ-8000; KEYENCE, Osaka, Japan). Cells were quantified using ImageJ software (Version 1.51, National Institutes of Health, Bethesda, MD, USA).

### Proliferation assay

ADSC proliferation was examined using a Cell Counting Kit-8 (Dojindo Laboratories, Kumamoto, Japan) in accordance with the manufacturer’s instructions. Briefly, 96-well transwell plates with a 0.4-μm pore membrane (3381; Corning) were used. ADSCs (1 × 10^4^ cells/well) or 10 ADSC spheroids/well (1 × 10^3^ ADSCs per spheroid) were seeded into the upper chambers in media supplemented with 10% FBS. Synovial fibroblasts (5 × 10^3^ cells/well) were seeded into the lower chambers and cultured in RPMI supplemented with 10% FBS. After 50 h at 37 °C, synovial cell proliferation in the lower chamber was evaluated. Optical density (OD) was measured at 450 nm using a Wallac 1420 ARVOsx Plate Reader (Perkin Elmer Life Sciences, Waltham, MA, USA).

### Apoptosis assay

Apoptosis in ADSCs was examined using a Caspase-Glo 3/7 Assay Kit (#G8090; Promega, Mannheim, Germany) in accordance with the manufacturer’s instructions. Briefly, ADSC single cells (1 × 10^4^ cells/well) or one ADSC spheroid/well (1 × 10^4^ ADSCs per spheroid) were seeded into white 96-well plates (#136101; Thermo Fisher Scientific, Waltham, MA, USA) in 100 μL DMEM with 1 μg/mL LPS without FBS and incubated for 24 h at 37 °C in 5% CO_2_. Media devoid of cells was set as control. Then, 100 μL Caspase-Glo 3/7 buffer was added to each well and equilibrated for 1 h at 20 °C. The absolute value of luminescence was measured, with luminescence defined as the level of apoptosis of target subjects (ADSC single cells and spheroids). The luminescence was measured at 24 h, 48 h, and 72 h after the baseline time, i.e., the time at which LPS was administered into the ADSCs.

### THP-1 cell culture and differentiation

The human acute monocytic leukaemia cell line, THP-1, was differentiated to macrophage according to a previous report^45^ with slight modifications^46,47^. Briefly, frozen THP-1 was obtained from the American Type Culture Collection (ATCC, Manassas, VA, USA). Then, after thawing, THP-1 was maintained at 37 °C with 5% CO_2_ in RPMI 1640 supplemented with 10% FBS, 2 mmol/L L-glutamine, 100 U/mL penicillin, and 100 mg/mL streptomycin. After medium change, THP-1 cells (5 × 10^5^/mL) were cultured in RPMI medium containing 200 nM phorbol 12-myristate 13-acetate (PMA, Sigma-Aldrich) in 24-well plates for 48 h. After incubation, cells were used for experiments as differentiated macrophages.

### Quantitative RT-PCR (RT-qPCR) analysis

Gene expression was evaluated in mouse ADSCs, human synovial cells, and THP-1 cells using RT-qPCR analysis. Human synovial cells isolated from RA patients were activated using 1 ng/mL *IL-1β* (Wako) for 24 h. Activated synovial cells (1 × 10^5^/well) in P4 were seeded onto 6-well plates with 1 × 10^5^ cells/well ADSC single cells in P4 or 1 × 10^2^/well ADSC spheroids (1 × 10^3^ cells in P4/spheroid) and cultured in RPMI supplemented with 10% FBS for 24 h at 37 °C. Cells were harvested and total RNA of ADSCs was extracted using an RNeasy Mini Kit (QIAGEN, Hilden, Germany). Total RNA of human synovial cells without activation was also extracted as control. Furthermore, cDNA was synthesized using a High-Capacity RNA-to-cDNA Kit (Thermo Fisher Scientific) and maintained at −30 °C for storage until PCR analysis. *TNF-stimulated gene-6* (*TSG-6*), *TGFβ1*, *IL-1β*, *TNFα*, and *IL-6* expression levels in ADSCs were evaluated using the primer sequences listed in Table 1. Expression levels were normalized to *GAPDH* levels.

**Table1:** Primer sequences of PCR for ADSC are shown.

Gene	Sequence (5′ to 3′)
GAPDH forward	AATGGTGAAGGTCGGTGT
GAPDH reverse	TGAAGGGGTCGTTGATG
TNF-α forward	ACCTTGTTGCCTCCTCCT
TNF-α reverse	GTTCAGTGATGTAGCGACAG
IL-6 forward	GATACCACTCCCAACAGA
IL-6 reverse	GCCATTGCACAACTCTTT
TSG-6 forward	GATACCCCATTGTGAAACCT
TSG-6 reverse	ACACCACCACACTCCTTTG
TGF-β1 forward	GACATCTCACACAGTATA
TGF-β1 reverse	GTTGCTATATTTCTGGTAG

Macrophages differentiated from THP-1 were stimulated using 10 ng/mL lipopolysaccharide (LPS) (Wako) for 24 h. Macrophages (1 × 10^5^ cells/well in P4) with and without stimulation were seeded onto 6-well plates and cultured with and without 1 × 10^5^ cells/well ADSC single cells in P4 or 1 × 10^2^/well ADSC spheroids (1 × 10^3^ cells in P4/spheroid) for 24 h at 37 °C in RPMI medium supplemented with 10% FBS. Cells were harvested at 24 h (before treatment) and 48 h (after treatment) and the total RNA of macrophages was extracted using a RNeasy Mini Kit (Qiagen). Then cDNA was synthesized and maintained at −30 °C for storage until PCR analysis. The expression levels of *TNF-α* and *IL-6* in macrophages under each condition were evaluated. Expression levels were normalized to *GAPDH* levels. All reactions were carried out using a 7500 Fast Real-time PCR system (Applied Biosystems/Thermo Fisher Scientific, Foster City, CA, USA) and SYBR green (SYBR Premix Ex Taq; Takara, Shiga, Japan) under the following conditions: 95 °C for 30 s, 40 cycles at 95 °C for 3 s, 55 °C for 30 s, and 72 °C for 30 s for TGFβ1 and 95 °C for 30 s, followed by 40 cycles at 95 °C for 3 s, and 60 °C for 30 s for all other genes. *GAPDH* was used as a control for cDNA quantity and quality. The threshold cycle (Ct) value, defined as the cycle number at which the fluorescence intensity exceeds the stipulated threshold, and comparative Ct were considered to evaluate the relative gene expression levels.

### Statistical analysis

Multiple-group differences were analysed using one-way analysis of variance, followed by the Bonferroni test for post-hoc analysis. p < 0.05 was considered statistically significant. Data were expressed as mean ± SEM (standard error of the mean). All p-values were two-sided. All analyses were performed using SPSS (Statistical Package for Social Science) Version 24 (IBM Corp., Armonk, NY, USA).

### Ethics statement

All methods pertaining to human subjects were carried out in accordance with the Declaration of Helsinki and the ethical guidelines and regulations for research involving human subjects of government. All methods involving animal subjects were carried out in accordance with the institutional guidelines and regulations for animal research. All human tissues and cells used in the experiments were collected from patients after obtaining informed consent.

All experimental protocols and ethical permission were approved by our institutional licensing research committee. (The approval number is 16017: Osaka City University Research Control Centre).

## Data Availability

The datasets used and/or analysed during the current study are available from the corresponding author on reasonable request.
