# Author Correction: Local transplantation of adipose-derived stem cells has a significant therapeutic effect in a mouse model of rheumatoid arthritis

**DOI:** 10.1038/s41598-022-10386-7

**Published:** 2022-04-15

**Authors:** Hideki Ueyama, Tadashi Okano, Kumi Orita, Kenji Mamoto, Masaaki Ii, Satoshi Sobajima, Hideki Iwaguro, Hiroaki Nakamura

**Affiliations:** 1 Department of Orthopedic Surgery, Osaka City University Graduate School of Medicine, Osaka, Japan; 2 Sobajima Clinic, Osaka, Japan; 3 Nanobridge LLC, Osaka, Japan

Correction to: *Scientific Reports* 10.1038/s41598-020-60041-2, published online 20 February 2020

Masaaki Ii was omitted from the author list in the original version of this article.

The Author Contributions section now reads:

H.U., T.O., K.M. and M.I. contributed to the experimental design. H.U. and T.O. wrote and edited the manuscript. H.U. and K.O. contributed to animal care, cell culture, and other experiments. M.I., S.S. and H.I. contributed clinical advice. H.N. supervised the study.

The original article has been corrected.

